# The Course of Schizophrenia Spectrum Disorders With Episodes of Catatonic Depressions

**DOI:** 10.1192/j.eurpsy.2024.1563

**Published:** 2024-08-27

**Authors:** A. Barkhatova, M. Bolgov, A. Smolnikova

**Affiliations:** Department of endogenous mental disorders and affective states, Mental health research center, Moscow, Russian Federation

## Abstract

**Introduction:**

Mood symptoms, especially depressive ones, occur in the majority of patients with schizophrenia spectrum disorders (SSD). Therefore, depression is often identified as one of the symptomatological dimensions of schizophrenia. Catatonia is also considered by some researchers as one of the dimensions of schizophrenia, or as an independent transnosological formation. Catatonia in SSD may be associated with affective dysregulation and is often accompanied by depression. Although the clinical course of SSD has been well studied previously, its relationship with psychopathological structure of episodes of SSD remains not entirely clear.

**Objectives:**

To determine the impact of episodes of catatonic depression on the course and prognosis of SSD.

**Methods:**

A sample of 60 patients with episodic course of SSD who met the criteria for catatonia according to the Bush-Francis Catatonia Screening Instrument (BFCSI) and for depression according to the Calgary depression schizophrenia scale (CDSS) was analyzed. An analysis of the clinical course of SSD was carried out on the basis of the medical history of all patients in the study sample and follow-up observation of 42 patients for 5 years. Global Assessment of Functioning Scale (GAF) was used to assess the prognosis of SSD.

**Results:**

Patients were divided into two groups depending on the period of manifestation of catatonia in the clinical course of SSD: during the first episode or during subsequent episodes. The sample of patients with the first episode (n=43, 71.7 %) was divided into three subgroups. A relatively favorable course of SSD was observed only in 13 patients (30.2 %; 21.7 % of SSD sample). The course of disorder was characterized by similar episodes with a high proportion of affective symptoms, long-term remissions and minimally expressed negative symptoms (GAF score=75.2±5.82). A relatively unfavorable course of SSD was observed in 15 patients (34.9 %; 25.0 % of SSD sample). It was characterized by moderate negative and chronic subdepressive symptoms with low frequency of catatonic and psychotic relapses (GAF score=62.3). An unfavorable course of SSD was also observed in 15 patients (34.9 %; 25.0 % of SSD sample). It was characterized by a high frequency of relapses with a tendency to form a chronic conditions with residual catatonic signs and psychotic symptoms (GAF score=50.1). In the sample of patients with manifestation of catatonia in the second or subsequent episodes (n=17; 28.3 %), the clinical course of SSD was unfavorable. It was characterized by a rapid augmenting of negative symptoms with the formation of psychomotor poverty syndrome with residual catatonic symptoms (GAF score=52.7).

**Conclusions:**

Our study shows that the occurrence of catatonic depressive episodes in the clinical course of SSD in most cases is an unfavorable prognostic factor.

**Disclosure of Interest:**

None Declared

